# Acknowledgement to Reviewers of *Foods* in 2018

**DOI:** 10.3390/foods8010032

**Published:** 2019-01-17

**Authors:** Foods Editorial Office

**Affiliations:** MDPI, St. Alban-Anlage 66, 4052 Basel, Switzerland

Rigorous peer-review is the corner-stone of high-quality academic publishing. The editorial team greatly appreciates the reviewers who contributed their knowledge and expertise to the journal’s editorial process over the past 12 months. In 2018, a total of 211 papers were published in the journal, with a median time to first decision of 15.8 days and a median time to publication of 35 days. The editors would like to express their sincere gratitude to the following reviewers for their cooperation and dedication in 2018:
Abete, Maria CesarinaMangia, Nicoletta PasqualinaAdjonu, RandyMarta, Ferreiro-GonzálezAguiar-Pinto Mina, Isabel Maria CravoMartinez, VicenteAlao, John PatrickMartínez, OlaiaAlbergamo, AmbroginaMarx, WolfgangAl-Busaidi, Moza A.Maugeri, AndreaAlexopoulos, AthanassiosMcAuley, Catherine M.Alvarez, CarlosMcComb, JacalynÁlvarez-Fernández, M. AntoniaMelini, ValentinaAndrade, María J.Merlino, Valentina MariaAngelino, DonatoMesías García, MartaAnnor, GeorgeMichalska, AnnaAsioli, DanieleMierzwa, DominikBai, JinheMiglietta, Pier PaoloBalakrishnan, GayathriMigliore, GiuseppinaBandyopadhyay, BidhanMilham, PaulBarclay, KateMiller, CharlesBarnych, BogdanMilovic, VladanBaumgartner, SabineMirzoian, ArmenBeasley, SheaMisciagna, GiovanniBebek, GayeMoran, LaraBeccaria, MarcoMoreira Martínez, Ramón FelipeBelenky, PeterMujaffar, SaheedaBerrios, José de JesúsMuniz, JuanBerthold-Pluta, AnnaMuñoz-Alférez, M. JoséBezirtzoglou, EugénieMuriana, PeterBisha, BledarMusalgaonkar, SharmishthaBohrer, Benjamin M.Muschiol, JanBonnaillie, Laetitia M.Muzzalupo, InnocenzoBosilevac, JosephNaik, SurabhiBosnea, LouloudaNambeesan, SavithriBožović, MijatNarsimhan, GanesanBraakhuis, AndreaNavarro-Alarcon, MiguelBrown, Amy C.Nochi, TomonoriBrownlee, Iain A.Noordergraaf, WimBuksa, KrzysztofNový, Ing. PavelBurris, KellieNúñez Carmona, EstefaníaButterworth, Peter J.Odeyemi, Olumide A.Cádiz-Gurrea, María LuzOhta, YoshijiCamire, Mary EllenOikonomou, Evdokia K.Campestre, CristinaOlejar, Kenneth J.Cardenia, VladimiroOlmedilla-Alonso, BegoñaCarvajal, MicaelaOracz, JoannaCarvalho, Ana P.Orfila, CarolineChang, Sheng-HsiungPadalkar, MugdhaChartier, AgnesPan, MeixiaChen, Lih-GeengPapadopoulou, ChrissanthyChen, ZhaoPapetti, AdeleChen, HuaiqiongParveen, IffatChiciudean, GabrielaPashikanti, SrinathChirumbolo, SalvatorePaulsen, PeterCho, SungeunPedrosa, Mercedes M.Christopoulos, M.V.Pérez-Álvarez, Jose AngelChuyen, Hoang V.Pérez-Lamela, ConcepciónColavita, GiampaoloPetrova, Olga E.Compagnone, DarioPetruk, GannaConte, LanfrancoPicozzi, ClaudiaCooksey, KayPinto, Carmelo GarcíaCosentino, CarloPłotka-Wasylka, JustynaCruz, Rui M.S.Prester, LjerkaD´Amico, StefanoPreti, RaffaellaD’Amico, MarioPurchas, Roger W.D’Archivio, MassimoPycia, KarolinaDean, LisaRaftopoulos, KonstantinosDegano, IlariaRagupathy, SubramanyamDermesonlouoglou, Efimia K.Raheem, BamideleDhillon, JaapnaRaja, Huzefa A.Dijkstra, GerardRaphaelides, StylianosDodds, ChrisReeve, BelindaDreher, MarkRen, Yuan DavidDuconseille, AnneReynolds, JoelDufossé, LaurentRicci, Elena ClaireDugo, GiacomoRijo, PatríciaDuke, ColinRincon, Angela M.Dykes, GaryRivera, TaniaElias, MiguelRodov, VictorEller, FredRodrigues, NunoErsan, SevcanRodríguez-Flores, M. ShantalEscolà-Gil, Joan CarlesRodríguez-López, PedroEstruch, RamonRoig-Sagués, ArturEtzel, MarkRosenthal, AndrewFakhr, MohamedRosicka-Kaczmarek, JustynaFang, ZhongxiangRoss, AlastairFaour-Klingbeil, DimaRoullier-Gall, ChloéFarzaneh, VahidRuiz-Capillas, ClaudiaFeke, Donald L.Rupasinghe, VasanthaFernandes, DonaldRutkowska, JaroslawaFernandes, José OliveiraRyan, Michael P.Ferrer-Gallego, RaulSaenz-Navajas, Maria-PilarFijan, SabinaSajdakowska, MartaFilteau, MarieSalaheen, SerajusFodor, MariettaSalles, ChristianFrancis, LeighSalvador, María DesamparadosFranzoi, MarcoSamborska, KatarzynaGan, Ren-YouSánchez-García, JavierGanesan, PalanivelSandell, MariGanzle, MichaelSaraiva, CristinaGarbowska, MonikaSatake, MasayukiGawlik-Dziki, UrszulaSayas, EstrellaGenovese, AlessandroSchmidtke, Leigh M.Gentry, TerrySenaadera, WijithaGhosh, AbhijitSenturk Parreidt, TugceGiacalone, DavideSeo, Woo DuckGiacometti, JasminkaSeratlic, SanjaGiannakourou, Maria C.Servili, MaurizioGiaouris, Efstathios D.Sevastianos, RoussosGimeno Alcañiz, José VicenteSharma, Girdhari M.Giuffrè, Angelo MariaShen, ZhenyuGolding, John B.Shih, Ming C.González-Domínguez, RaúlShinomiya, KazufusaGradeci, KlodianSiano, FrancescoGrafenauer, SaraSicari, VincenzoGraikou, KonstantiaSimal-Gandara, JesusGrech, AmandaSingh, Anubhav PratapGroh, Isabel Anna MariaSingh, PrashantGrosso, GiuseppeSirsat, SujataGueimonde, MiquelSmutzer, GregoryGuerrero, Raul F.Soares Silva, Isabel JoaoHackett, MarkSobeh, MansourHaldar, SumantoSoglia, FrancescaHarrington, Robert J.Sontag-Strohm, TuulaHarrison, SabineSørensen, Jens LauridsHauge, Sigrun J.Sørensen, Klavs MartinHefni, MohammedSpagnuolo, RoccoHernández, FranciscaStadnik, JoannaHill, Arthur R.Stan, LauraHorner, Harry T.Steup, MartinHsieh, ChangweiStote, KimHungerford, JamesStrati, Irini F.Hurtaud, CatherineSugiyama, KemmyoJimenez-Flores, RafaelSummo, CarmineKaddoumi, AmalSwieca, MichałKamiloglu, SenemSzeleszczuk, ŁukaszKandylis, PanagiotisTappi, SilviaKarayannakidis, PanayotisTaylor, MatthewKaripidis, PhilipposTepper, Beverly J.Karoglan, MarkoTessier, Frederic JKassama, LaminThomareis, ApostolosKatayama, ShigeruTirloni, EricaKelly, MatthewTixier, Anne-SylvieKelly, AlanTremblay, AngeloKeyzers, Robert A.Trijp, Hans VanKhallouki, FaridTripodi, GianlucaKhouryieh, HannaTsai, Mei-LinKilcawley, KieranTudela, JoséKilleen, DanielTunick, MichaelKim, Mina K.Tylewicz, UrszulaKlempt, MartinUribarri, JaimeKöhler, KarstenVachali, PreejithKoutelidakis, Antonios E.Valdes García, ArantzazuKrishnaswamy, KirubaVan Ballegooijen, HanneKulig, DominikaVan der Kamp, Jan WillemKulis, MichaelVan Herck, Mikhaïl A.Kurek, MarcinVargas, LilianaKus, PiotrVemuri, RavichandraKuzma, LukaszVenturi, FrancescaKwon, OranVerardo, VitoLabra, MassimoVerma, AnilLadero, VictorVinceti, MarcoLai, YandongVogt, Leonie M.Laming, Donald Richard JohnVyviurska, OlgaLanza, MassimilianoW.M.Leung, DavidLawrence, MarkWaehrens, Sandra S.Lee, Young SeongWahab, M. FarooqLegeza, BalázsWang, FanLeong, Thomas Seak HouWang, GuorongLeong, Sze YingWardenaar, FlorisLi, Fu-ShuangWelch, Aaron Z.Lim, Beong OuWeller, PhilippLivesey, GeoffreyWiesław, WiczkowskiLoizzo, Monica R.Winson, AnthonyLopez, GabrielaWojdyło, AnetaLücke, Friedrich-KarlWojnowski, WojciechLundén, JanneWójtowicz, AgnieszkaLung, Ming-YeouWu, Wen-TzuMaeda, HayatoXu, YongMagalhães, Júlia M.C.S.Yamaki, KohjiMahapatra, Ajit K.Yeoman, CarlMaleszka, AlicjaZanzer, Yoghatama CindyaMalissiova, EleniZhang, Yue

